# Clinical Factors Predicting Disseminated Intravascular Coagulation (DIC) in Women With Placental Abruption and a Live Fetus

**DOI:** 10.7759/cureus.42506

**Published:** 2023-07-26

**Authors:** Miwa Miyazaki, Shunji Suzuki

**Affiliations:** 1 Obstetrics and Gynecology, Japanese Red Cross Katsushika Maternity Hospital, Tokyo, JPN; 2 Obstetrics and Gynecology, Nippon Medical School, Tokyo, JPN

**Keywords:** neonatal asphyxia, surviving fetus, placental abruption, disseminated intravascular coagulation (dic), predicting factors

## Abstract

Objective: We examined predicting factors other than blood test results for disseminated intravascular coagulation (DIC) in patients with placental abruption and a live fetus who were transported by ambulance to our institute.

Methods: We reviewed the obstetric records of 60 singleton deliveries between January 2006 and December 2018. In this study, we excluded four cases with fetal demise at the time of transportation. In the other 56 cases, therefore, emergency cesarean section was performed at the time of diagnosis of placental abruption. Of the 56 cases, 12 cases were complicated by DIC (21.4%). Therefore, clinical risk factors leading to DIC other than intrauterine fetal demise (IUFD) were retrospectively examined with the remaining 44 cases set as control (78.6%).

Results: In evaluation with multivariate analysis, severe neonatal asphyxia (neonatal Apgar score <4 at 1 minute: adjusted odds ratio 2.89, p <0.01 and umbilical artery pH <7: adjusted odds ratio 4.01, p <0.01) was an independent risk factor for DIC, while short time interval from the onset to delivery (<1 hour; adjusted odds ratio 0.195, p = 0.04) was an independent negative risk factor for DIC.

Conclusion: Severe neonatal asphyxia was a risk factor for DIC in cases of placental abruption in those transported by ambulance with surviving singleton fetuses, while a short time interval from the onset to delivery was a negative risk factor for DIC.

## Introduction

Placental abruption is one of the serious causes of visits to the emergency department and a major reason for intrauterine fetal demise (IUFD)/neonatal asphyxia and maternal disseminated intravascular coagulation (DIC) during pregnancy [1,2]. Placental abruption with IUFD has been reported to be associated with a high risk of postpartum hemorrhage resulting from severe DIC [1,2]. In cases of placenta abruption with IUFD, continuous monitoring is indispensable for identifying progressive fibrinogen reductions after delivery of the dead fetus.

In the case of placental abruption who was transported by ambulance, when fetal survival is confirmed, prompt fetal delivery is required, and there is no time to wait for the results of a maternal blood test; however, it is possible to experience cases leading to DIC after the delivery of a living fetus with placental abruption, although the incidence seems to be lower than that in the cases with fetal demise.

Based on these backgrounds, we examined predicting factors other than blood test results for DIC in cases of placental abruption with a live fetus transported by ambulance to our institute.

## Materials and methods

The protocol for this study was approved by the Ethics Committee of the Japanese Red Cross Katsushika Maternity Hospital. In addition, informed consent concerning retrospective analysis was obtained from each subject during their hospital visit.

We reviewed the obstetric records of singleton deliveries complicated by placental abruption developed outside our institute and transported to our institute by ambulance (n = 60), defined as the findings of separation of a normally implanted placenta indicated by evidence of retro-placental hemorrhage after 22 weeks of gestation between January 2006 and December 2018. In this study, we excluded four cases with fetal demise at the time of transportation. Our institutional policy is to prioritize prompt delivery over performing blood tests if the fetus is alive with apparent placental abruption. In the other 56 cases, therefore, an emergency cesarean section was performed at the time of diagnosis of placental abruption because the cervix was not fully dilated. Instead, blood tests were performed in all cases at the end of the cesarean section. All cases were diagnosed as placental abruption by ultrasonography at the emergency department and confirmed by operative findings macroscopically. In all cases, the time from the diagnosis to the start of the cesarean section was less than 10 minutes.

In this study, DIC was diagnosed using the obstetric DIC score approved by the Japanese Society of Obstetrics and Gynecology or the DIC score of the Japanese Association for Acute Medicine [3,4]. In detail, the obstetric DIC score has three components: (1) the underlying diseases, (2) the clinical symptoms, and (3) the laboratory findings (coagulation tests) [3]. If the score reaches 8 points or more, obstetric DIC is diagnosed. In addition, higher scores are given for clinical parameters rather than for laboratory parameters. Therefore, it can help with making a prompt diagnosis and starting treatment early for DIC.

In this study, of the four cases with IUFD, all cases were complicated by DIC. Of the other 56 cases, 12 cases were complicated by DIC (21.4%, DOC group). Therefore, in this study, clinical risk factors leading to DIC other than IUFD were retrospectively examined with the remaining 44 cases set as the control group (78.6%).

According to some previous studies concerning placental abruption and DIC [1,2,5-10], we examined the maternal age, parity, gestational week at delivery, the prevalence of hypertensive disorders at delivery, presence or absence of external bleeding, premature rupture of the membranes, the time interval from the onset of the symptoms of placental abruption to delivery, estimated total blood loss from the onset to the end of the cesarean section, neonatal birth weight, an Apgar score of <4 at 1 and 5 minutes, and an umbilical artery pH of <7. Hypertensive disorders were defined as blood pressure ≥ 140/90 mmHg measured on two or more occasions at least six hours apart with the patient at rest. The onset was defined as the time when symptoms (abdominal pain and/or external bleeding) due to placental abruption appeared, as assessed retrospectively. Small-for-gestational-age infants were defined according to the neonatal birth weight standards for gestational age in Japan [11].

Data are presented as numbers (%). The statistical software SAS version 8.02 (SAS Institute, Cary, NC, USA) was used for statistical analyses. The Χ2 test for categorical variables was used for statistical analysis. Differences with p <0.05 were considered significant. Logistic regression was then performed to identify the risk factors strongly associated with the occurrence of DIC in a multivariate model. Variables used in the multivariate model were those that had shown a significant (p <0.05) association with the occurrence of DIC on univariate analysis. ORs and 95% CIs were also calculated.

## Results

Table 1 shows the clinical characteristics of patients complicated by placental abruption with surviving singleton fetuses with and without DIC. The incidence of DIC in the patients complicated by placental abruption with surviving singleton fetuses was significantly higher in those of maternal age ≥35 years (p = 0.02), preterm delivery (p = 0.03), and severe neonatal asphyxia (p <0.01). While the incidence was significantly lower in the presence of external bleeding (p < 0.01) and time interval from the onset to delivery ≤1 hour (p = 0.04).

**Table 1:** Clinical characteristics of patients complicated by placental abruption with surviving singleton fetuses with and without DIC DIC: disseminated intravascular coagulation

	Control group (n = 44)	DIC group (n = 12)	P-value
Maternal age ≥35 years	13 (30)	8 (67)	0.02
Nulliparity	31 (70)	6 (50)	0.19
Gestational age at delivery <32 weeks	5 (11)	5 (42)	0.02
Gestational age at delivery <37 weeks	31 (70)	12 (100)	0.03
Hypertensive disorders	2 (5)	2 (17)	0.18
Presence of external bleeding	29 (66)	2 (17)	<0.01
Premature rupture of the membranes	13 (30)	1 (8)	0.13
Time interval from the onset to delivery <1 hour	12 (27)	0	0.04
Time interval from the onset to delivery <3 hour	27 (61)	8 (67)	0.74
Time interval from the onset to delivery <5 hour	43 (98)	12 (100)	0.60
Estimated total blood loss from the onset to delivery ≥1000 mL	21 (48)	9 (75)	0.09
Estimated total blood loss from the onset to delivery ≥2000 mL	0	0	1
Small-for-gestational-age infants	2 (5)	1 (8)	0.61
Neonatal Apgar score <4 at 1 min	7 (16)	9 (75)	<0.01
Neonatal Apgar score <4 at 5 min	1 (2)	3 (25)	<0.01
Umbilical artery pH <7	4 (9)	8 (67)	<0.01

As shown in Table 2, in evaluation with multivariate analysis using the nine potential risk factors as shown in Table 1, severe neonatal asphyxia (neonatal Apgar score <4 at 1 minute and umbilical artery pH <7) was an independent risk factor for DIC, while short time interval from the onset to delivery (<1 hour) was an independent negative risk factor for DIC.

**Table 2:** Association between potential risk factors and the occurrence of DIC in cases of placental abruption with surviving singleton fetus by multiple logistic regression DIC disseminated intravascular coagulation, OR: odds ratio, 95% CI: 95% confidence interval

	Adjusted OR	95% CI	P-value
Maternal age ≥35 years	3.85	0.99-14	0.08
Gestational age at delivery <32 weeks	2.86	0.67-12	0.06
Gestational age at delivery <37 weeks	1.16	0.89-1.2	0.11
Presence of external bleeding	0.51	0.12-1.4	0.12
Premature rupture of the membranes	0.26	0.071-1.2	0.08
Time interval from the onset to delivery <1 hour	0.195	0.042-0.84	0.04
Neonatal Apgar score <4 at 1 min	2.89	1.4-4.7	<0.01
Neonatal Apgar score <4 at 5 min	5.85	0.81-23	0.06
Umbilical artery pH <7	4.01	1.6-9.3	<0.01

## Discussion

In this study, severe neonatal asphyxia was a risk factor for DIC, while a short time interval from the onset to delivery was a negative risk factor for DIC in cases of placental abruption transported by ambulance with surviving singleton fetus.

The former may be the same mechanism as previous reports indicating the association between the incidence of IUFD and DIC following placental abruption [1,2]. It is defined as a severe abruption when a placental abruption is complicated by IUFD or severe fetal/neonatal asphyxia [1,2,12]. It has been observed that patients who develop placental abruption with IUFD suffer from a combined coagulopathy that combines consumption-type coagulopathy and massive release of thromboplastin into the maternal systemic circulation [13,14]. In addition, local hypoxia and hypovolemia may cause serious endothelial response. In patients under these conditions, more blood loss, decreased fibrinogen levels and platelet counts, and elevated levels of fibrin degradation products, D-dimer, and prothrombin time have been observed compared to those in patients without these conditions [2]. In addition, in these cases, there may be normal fibrinogen levels at the time of cesarean section but later displayed markedly decreased fibrinogen levels.

Regarding the latter, some similar previous observations have been found [7,15]. The impact of the abruption severity and the onset-to-delivery time on the maternal and neonatal outcomes of cases of clinically diagnosed placental abruption have been well examined [7]. For example, the range of placental separation has been observed to be correlated with neonatal outcomes, and there has been a significant negative correlation between the onset-to-delivery time and the umbilical artery pH in moderate abruption [7]. In our previous study, adverse outcomes due to placental abruption which developed at home seemed to be associated with the symptom of pain without bleeding and a long time interval between the onset and hospital (or clinic) visit [14]. Therefore, the current results will support those of previous studies [7,15], and we should reiterate the importance of recognizing the early symptoms of placental abruption in Japanese pregnant women [16].

We understand the presence of serious limitations. First, the sample size is very small. Second, in this study, we gave priority to saving the life of the fetus and started cesarean section within 10 minutes from the diagnosis of placenta abruption in all cases as clinicians; however, if the results of blood tests could be obtained quickly, blood transfusion could have been started earlier, and the progression to DIC may be prevented [6]. We have no intention of changing our current policy as long as we cannot make a blood transfusion decision based on the results of the blood test within minutes. In addition, in our previous study, placental implantation is related to the severity of placental abruption [17]; however, this study did not examine this point. For example, in cases showing placental implantation on the posterior wall, it may be difficult to observe placental conditions behind the fetus on ultrasonography. In addition, the abruption in cases of placenta implanted posteriorly may be less painful; however, the fetus and mother may be at risk [18]. Unfortunately, in these cases, the position of the placenta was not well documented as the fetus may be moribund.

## Conclusions

Severe neonatal asphyxia was a risk factor for DIC in cases of placental abruption transported by ambulance with surviving singleton fetuses, while a short time interval from the onset to delivery was a negative risk factor for DIC. Therefore, maternal management should be carefully monitored for the development of DIC in severe neonatal asphyxia and long time interval from the onset to delivery.
